# The potential use of mesenchymal stem cells and their exosomes in Parkinson’s disease treatment

**DOI:** 10.1186/s13287-022-03050-4

**Published:** 2022-07-28

**Authors:** Reza Mosaddeghi Heris, Milad Shirvaliloo, Sanaz Abbaspour-Aghdam, Ali Hazrati, Ali Shariati, Hamed Rahmani Youshanlouei, Farhad Jadidi Niaragh, Hamed Valizadeh, Majid Ahmadi

**Affiliations:** 1 Neurosciences Research Center, Tabriz University of Medical Sciences, Tabriz, Iran; 2 Infectious and Tropical Diseases Research Center, Tabriz University of Medical Sciences, Tabriz, Iran; 3 Stem Cell Research Center, Tabriz University of Medical Sciences, Tabriz, Iran; 4 Department of Immunology, Faculty of Medical Sciences, Tarbiat Modares University, Tehran, Iran; 5 Faculty of Medicine, Tabriz University of Medical Sciences, Tabriz, Iran; 6 Department of Immunology, School of Medicine, Tabriz University of Medical Sciences, Tabriz, Iran; 7 Tuberculosis and Lung Disease Research Center, Tabriz University of Medical Sciences, Tabriz, Iran

**Keywords:** Mesenchymal stem cell, Exosome, Parkinson's disease, Therapeutic application

## Abstract

Parkinson's disease (PD) is the second most predominant neurodegenerative disease worldwide. It is recognized clinically by severe complications in motor function caused by progressive degeneration of dopaminergic neurons (DAn) and dopamine depletion. As the current standard of treatment is focused on alleviating symptoms through Levodopa, developing neuroprotective techniques is critical for adopting a more pathology-oriented therapeutic approach. Regenerative cell therapy has provided us with an unrivalled platform for evaluating potentially effective novel methods for treating neurodegenerative illnesses over the last two decades. Mesenchymal stem cells (MSCs) are most promising, as they can differentiate into dopaminergic neurons and produce neurotrophic substances. The precise process by which stem cells repair neuronal injury is unknown, and MSC-derived exosomes are suggested to be responsible for a significant portion of such effects. The present review discusses the application of mesenchymal stem cells and MSC-derived exosomes in PD treatment.

## Introduction

PD occurs as a result of dopamine depletion, loss of neurons in substantia nigra (SN), and Lewy body build-up in other brain areas [1–3]. Patients with PD experience bradykinesia, stiffness, tremors at rest and unsteady gait as their motor abilities deteriorate [2, 3]. Deep brain stimulation or therapies to increase DA levels by administering a DA precursor (Levodopa) are the currently available therapy options for PD. However, L-DOPA therapy has little effect on the progression of PD, and its efficacy decreases as the disease progresses. At the same time, undesirable symptoms such as dyskinesia might also occur [4–6]. In vitro studies have shown that stem cells can differentiate into DAn [7–12] and accelerate the recovery of injured DAn [13–17].

Stem cells can be classified according to their origin: hematopoietic stem cells, neural stem cells, epithelial stem cells, skin stem cells, mesenchymal stem cells (MSCs), embryonic stem cells (ESCs), induced pluripotent stem cells (iPSCs), and neural stem cells (NSCs) [18]. MSCs can be originated from different sources, including adult bone marrow, adipose tissue, peripheral blood and various neonatal birth-associated tissues and they can also be induced in vitro to differentiate into osteoblasts, chondrocytes, adipocytes, and other cell types [19]. MSCs are multipotent non-hematopoietic cells with certain advantages over others. MSCs have a minimal immunogenicity, no risk of teratoma, and no ethical issues. Another advantage is their potential for individualized therapy, as MSCs can be obtained from the same patients without triggering immunological reactions. Furthermore, MSCs have a low risk of tumorigenesis after transplantation into humans or animals [18]. However, recent investigations have suggested that the paracrine actions of MSCs are mediated by the formation and release of extracellular vesicles (EVs) [20]. Exosomes are a subtype of EVs that range from 30 to 150 nm in size and are secreted by all living organisms cells [21, 22]. MSCs and their exosomes have been used in several clinical studies around the world to treat a variety of diseases, such as bone and cartilage pathologies[23], diabetes, cardiovascular disease, immune-related and neurological problems, as well as diseases of the lungs, kidneys, and liver [24–26]. This therapeutic technique may be beneficial for presently incurable diseases. Still, important uncertainties remain about the administration routes, ideal MSC dosage, best engraftment period, and the destiny of the cells being infused [27]. In the present article, we will focus on the therapeutic potential of MSCs and their exosomes in treating PD.

## Pathophysiology of PD

PD is a degenerative disease that affects 0.3% of the population and causes progressive impairment in motor function [28]. Misfolded proteins, such as a-synuclein, as a possible pathologic agent, accumulate abnormally in the brain [29], causing Lewy body dementia, PD dementia, multisystemic atrophy and PD.

Toxic a-synuclein oligomers and protofibrils [30] can be transmitted from one cell to another in a prion-like way [31]. This is thought to facilitate the pathogenesis of PD, as a-synuclein oligomers are known to spread from the basal to the neocortical parts of the brain [32]. In addition to a-synuclein build-up, an amyloid-beta and tau containing a-synuclein coaggregation were recently discovered [33–35]. Specific mechanisms underlying neuronal degeneration in PD are yet to be identified. However, infectious agents [36], pesticides [37], heavy metals [38], and rural life [39] have been recognized as risk factors for PD.

Although environmental risk factors for Parkinson's disease have attracted a lot of attention, the role of genetic variables in determining the possibility of having the disease is becoming more widely realized. Although familial types of PD account for less than 10% of all cases, the identification of multiple genes (e.g., *parkin*, *DJ1*[a parkin-associated protein involved with oxidative stress], *α-synuclein*, *UCHL1* [ubiquitin carboxy-terminal hydrolase L1], and *PINK1* [putative serine-threonine kinase]) that cause early onset PD has provided vital information about possible pathological pathways [40]. Many other genes have been linked to the parkinsonian phenotype; however, neurological testing frequently reveals other signs such as ataxia, dystonia, or dementia [41].

The pathogenesis of PD is influenced by cell metabolism and protein clearance. Degeneration of noradrenergic neurons in the locus coeruleus could be associated with depression and dementia [42]. Depression can also be caused by the degeneration of serotonergic neurons in the median raphe and raphe obscurus [43].

## Modulatory effects of MSCs on PD

Recently, dysregulation of the autophagy system has been identified in the brains of PD patients and animal models of the condition, suggesting a potential role for autophagy in PD [44]. In PD models, MSCs have been demonstrated to improve a-syn clearance and regulate autophagy-lysosomal activity [45]. MSCs may activate autophagy signaling through upregulation of Beclin-1 [46], a key positive regulator of mammalian autophagy. The secretome of MSCs has been found to contain numerous components associated with autophagy signaling in cell-based experiments through induction of autophagy-related genes, including beclin-1 *(BCEN1)*, Gamma-aminobutyric acid receptor-associated protein-like 1 *(GABARAPL1)* and Autophagy related 12 *(ATG12)*. The secretome of MSCs drives PI3K/Akt activation and modulates different signaling pathways to improve nutrient absorption, cell growth, metabolism, and proliferation [47, 48].

According to several investigations, MSCs exhibit immunomodulatory effects after infiltrating to injury sites in response to particular chemotactic recruitment [49] and releasing numerous growth and immunoregulatory factors, so they can alleviate inflammation and improve tissue healing [50]. Therefore, MSC-based cell therapy has been used to modulate inflammation and accommodate tissue regeneration in treating many neuroinflammatory and neurodegenerative illnesses such as Parkinson's disease [51].

MSCs are also believed to have immunoregulatory effects since they might induce inflammation when the immune system is underactive and suppress inflammation when the immune system is overactive. This process is often referred to as the immune system's "sensor and switcher" [52]. In animal models of epilepsy and PD, MSC therapy increased anti-inflammatory cytokine levels like Transforming growth factor-beta1 (TGF-β1), Prostaglandin E2 (PGE2), Hepatocyte growth factor (HGF), Indoleamine 2,3 dioxygenase (IDO), Nitric oxide (NO), interleukin 4 (IL-4) and interleukin 10 (IL-10), while decreasing pro-inflammatory cytokine levels (such as interleukin-6 (IL-6), Interleukin-1beta (IL-1β), Tumor necrosis factor-alpha (TNF-α)) in the brain and blood[53–56]. Consistent with these findings, it has been proposed that TNF promotes the immunosuppression capacity of MSCs by increasing the expression of TNF-α stimulated gene/protein-6 (*TSG-6*) to limit microglial activation (56) effectively. More importantly, an investigation found that MSCs might alter local microglia from a pro-inflammatory state to an anti-inflammatory state via the CX3CL1/CX3CR1 signaling axis [57].

On the other hand, some therapeutic effects appear to be dependent on MSC-released neurotrophic factors (Glial cell-derived neurotrophic factor (GDNF), Nerve growth factor (NGF), Brain-derived neurotrophic factor (BDNF)), which can inhibit DA neurons apoptosis and enhance neurogenesis by secreting proangiogenic and mitotic factors like Vascular endothelial growth factor (VEGF) and, fibroblast growth factor 2 (FGF2) [58, 59].

## Preclinical studies of MSC therapy on PD

### Bone marrow-derived MSCs

Recently Bouchez et al. used rat bone marrow mesenchymal stem cells (BMSCs) to treat PD in animal models. In this study, rat BMSCs were administered to the same location in adult rats that had been injured by unilateral striatal injection of 6-hydroxydopamine (6-OHDA) [15]. The frequency of amphetamine-induced rotations, caused by the pre-synaptic positive regulatory effect of amphetamine on synaptic concentrations of dopamine, was dramatically reduced in transplanted rats compared to the control group. This test evaluate the motor impairment induced by lesions in PD disease [60].The level of dopaminergic biomarkers in nerve endings and cell bodies was largely recovered. This effect was attributed to the survival of dopaminergic neurons and budding from the remaining nigrostriatal fibers.

In a 6-OHDA animal model, intracarotid injection of rat BMSCs was tested by Cerri et al. [61]. While there was no change in the progression of 6-OHDA-induced lesion or motor impairment, there was a gradual normalization of the pathologic reaction to apomorphine treatment. The authors stated that arterial administration of BMSCs might result in functional compensatory modifications in the nigrostriatal pathway, but neuroprotective effects remain unknown.

Intravenous (IV) delivery of rat BMSCs was investigated in another animal model of PD created by subcutaneous (SC) administrations of rotenone and ovariectomy [62]. Ahmed et al. demonstrated that BMSCs were capable of spreading in the damaged brain and resulted in a significant drop in serum TGF-1 concentrations, as well as an elevation in nestin gene expression and brain tyrosine hydroxylase (TH). DA levels in the brain and serum BDNF were likewise raised. The anti-inflammatory, immunomodulatory, and neurotrophic actions of BMSCs are thought to be responsible for normalizing these indicators. The striatum's histologic integrity was also preserved in the brain slices [62].

To achieve functional benefits, the appropriate dosage and delivery rate will be better identified in future investigations. Intranasal administration of murine BMSCs was also evaluated in a rat model created by intraperitoneal (IP) rotenone administrations [63]. Histological analysis in various brain areas confirmed the successful survival of MSCs. Nigral dopaminergic neurons and striatal TH-positive fibers were retained considerably. Furthermore, neurobehavioral testing revealed that MSC treatment could slow the gradual rotenone-induced loss of locomotor capabilities [57].

### Adipose tissue-derived MSCs

In another study by Schwerk et al., seven days after the medial forebrain bundle was entirely damaged by administrations of 6-OHDA, autologous adipose tissue-derived mesenchymal stem cells (AD-MSCs) were grafted into the substantia nigra of rats [64]. Compared to untreated animals, enhanced subventricular zone (SVZ) neurogenesis was observed three days following transplantation. This observation is intriguing because impairment of the SVZ-olfactory bulb pathway is believed to be the etiology of hyposmia in PD patients. Transplanted AD-MSCs were predominantly seen in the SN area and the adjacent arachnoid mater. Some cells with endothelial characteristics were also discovered adjacent to arteries. This line of differentiation could be effective in amending vascular changes (Table 1).

**Table 1:** MSC-based preclinical studies in PD treatment

Experimental model	Transplantation method	Results and conclusion	References
Rats were lesioned by stereotactic injection of 6-OHDA into the right medial forebrain bundle	Stereotaxic method	Because of the dopamine function, CJ-MSCs therapy may protect against PD problems and nerve induction of cells. CJ-MSCs microencapsulation, on the other hand, leads to an even greater protective impact of CJ-MSCs	[65]
30 B57BL/6 mice were lesioned through the intraperitoneal administration of rotenone	Intranasal delivery (IN)	The degeneration caused by rotenone therapy was considerably reversed in mice receiving IN administration of MSCs, demonstrating that IN delivery of MSCs may be a possible safe, straightforward, and cheap alternative method for stem cell treatment in neurodegenerative illnesses	[63]
24 adult female Sprague–Dawley rats were subcutaneously injected with rotenone	Intravenously	The lesioned brains were able to attract BM-MSCs, which resulted in a large drop in serum TGF-1 and monocyte chemoattractant protein-1 (MCP-1) levels, as well as a considerable elevation in serum BDNF and brain DA levels, as well as brain TH and nestin gene expression. Furthermore, treatment with BM-MSCs preserved the histological structure of the striatum in brain slices	[62]
Male Wistar rats lesioned by 6-OHDA intrastriatal injection	Intraarterially	Infusion of rat MSCs had no effect on the progression of 6-OHDA-induced damage or motor impairment during the stepping test, but it did cause progressive normalization of the pathological response (contralateral turning) to apomorphine treatment	[61]
Adult male Wistar rats were lesioned by stereotactic injection of 6-OHDA into the right medial forebrain bundle	Intranigral injection	Subventricular neurogenesis was significantly higher in MSC-transplanted rats compared to non-transplanted animals three days after transplantation. The majority of MSC were discovered in the substantia nigra and adjacent arachnoid mater, expressing S100b and brain-derived neurotrophic factor, whereas some MSC had an endothelial phenotype and were located near blood vessels	[54]
40 adult male Wistar rats received 2 μl 6-OHDA infusions into the medial forebrain bundle	Intranigral infusion	MSC expressing pericyte and endothelial markers were found around the substantia nigra and the arachnoid mater. MSCs increased peripheral antiinflammatory cytokines while preserving dopamine levels. In addition, adipose-derived MSC improved memory performance by increasing neurogenesis in the hippocampus and subventricular areas	[64]
Female Sprague–Dawley rats were lesioned by a single stereotaxic injection of 6-OHDA into the left medial forebrain bundle	Injection into the striatum	In this pilot study, umbilical cord matrix stem (UCMS) cells reduced apomorphine-induced rotations. Brain tumors, rotating behavior, or a frank host immune rejection response were not observed after UCMS cells were transplanted into normal rats	[66]
Rats were lesioned through the unilateral rotenone injection of the ventral tegmental area (VTA) and the SNc	Intrastriatal infusion	HUMSC transplantation reduced apomorphine-evoked rotations and dopaminergic neuron loss in the lesioned SNc, which was greatly aided by VEGF expression in HUMSCs. This paper discusses the feasibility of HUMSC as a vector for gene therapy and argues that stem cell engineering with VEGF may improve the transplantation technique for Parkinson's disease treatment	[67]
Adult female Sprague–Dawley rats were lesioned by unilateral intra-striatal injection of 6-OHDA	Intrastriatal infusion	In a rat model of PD, transplantation of a population of adult bone-marrow MSC induced partially in a neural pathway restores in part the dopaminergic function of the nigrostriatal pathway, leading to an early improvement in behavior, an increased density of dopaminergic markers, and an in vivo recovery of DA release	[15]

In their prospective study [54], Schwerk et al. reported the presence of AD-MSCs near the arachnoid mater and the SN, where they expressed pericyte and endothelial biomarkers. Improved memory performance, upregulation of systemic anti-inflammatory cytokines, conserved levels of dopamine, and enhanced neurogenesis in hippocampus and SVZ were attained following transplantation of AD-MSCs. However, improvements in cognitive functions were not matched by gains in motor abilities.

### Human dental pulp-derived MSCs

Human dental pulp stem cells (h-DPMSCs) have also yielded promising results, according to Chun et al. experiments in vitro [68]. It has been shown that DPMSCs can develop into neural lines when cultivated in specific conditions, and an increase in TH expression was also found in some of these cells.

### Human conjunctiva-derived MSCs

Another in vivo study on the rat model of PD by Forouzandeh et al. found that MSCs obtained from human conjunctiva (CJ-MSCs) exhibited protective effects against PD sequelae and nerve induction of cells owing to their capability to release dopamine. On the other hand, CJ-MSC microencapsulation leads to an even more significant protective impact of CJ-MSCs [65].

## Clinical trials of MSC therapy on PD

### Bone marrow-derived MSCs

Autologous BMSCs were grafted into the SVZ of seven patients with severe PD in a prospective, uncontrolled, pilot study by Venkataramana in 2010 [69]. This brain area was selected because it possesses the greatest amount of NSCs participating in neurogenesis. The Unified PD Rating Scale (UPDRS), Schwab and England (S&E), and Hoehn and Yahr (H&Y) scales were used to assess clinical circumstances throughout a 10–36 month period. Symptoms like facial expression, gait, and freezing periods were also reported to have improved subjectively. Furthermore, in two cases, the L-DOPA doses were dramatically lowered. Although the particular mechanisms are unknown, this experiment has validated the procedure's safety, as none of the patients experienced clinical exacerbation until the completion of the monitoring period. Furthermore, there were no aberrant parenchymal alterations on cerebral magnetic resonance imaging [69].

Furthermore, modified MSCs that had been differentiated into dopaminergic cells were directly inserted into the femoral artery that supplies the substantia nigra in a recent trial on 53 PD patients [70]. The findings demonstrate that intra-arterial autologous BMSCs transplantation is a safe and effective therapy and eliminates the tumorigenesis and immunological reactions risks. This is supported by the fact that none of the patients treated with this method developed severe adverse complications, and as the study suggests, all participants were able to leave the hospital the day after receiving MSC infusion.

In another pilot study by Canesi et al., five patients with progressive supranuclear palsy, an uncommon and severe form of parkinsonism, were given bone marrow-derived MSCs by administration into the cerebral arteries [71]. All treated patients survived a year after cell infusion, except one, who died nine months later for causes unrelated to cell administration or progression of the disease (accidental fall). During the one-year follow-up, motor function assessment scales in all treated individuals remained steady for at least six months. In late 2021, Schiess et al. confirmed the safety and tolerance of intravenous administration of bone marrow-derived MSCs on 20 subjects with mild to moderate PD, who exhibited reduced UPDRS scores by the end of a one-year follow-up interval [72].

### Human umbilical cord-derived MSCs

Ever since Venkataramana in 2010 [69], several other investigations have been conducted to confirm the efficacy of MSC therapy on PD using the scales mentioned above. In 2011, Qiu et al. performed human umbilical cord-derived MSC (hUC-MSC) transplantation on 8 patients with PD, observing a clinical improvement in UPDRS within a month from transplantation [73]. Three years later, in 2014, Wang et al. enrolled 15 patients with PD (H&Y staging: 3 – 5) into an experimental program on hUC-MSC transplantation, demonstrating a significant decreased in UPDRS within a month after the intervention [74]. In 2016, similar results were reported by a limited trial on intravenous transplantation of allograft hUC-MSCs in 5 PD cases, that led to reduced disease severity in 3 out of the 5 patients, based on UPDRS, in three months [75]. More recently, in 2020, Boika et al. explored the efficacy of autologous MSC transplantation on 12 PD patients. The therapy's effectiveness was assessed one and three months after the transplant. UPDRS was used to determine the severity of motor symptoms. In the post-transplant period, they discovered a statistically significant reduction in the severity of motor and nonmotor complaints in the study participants [76].

### Adipose tissue-derived MSCs

Most recently, in early 2022, Shigematsu et al. treated 3 PD patients with 5 – 6 repeated infusions of autologous adipose tissue-derived MSCs, denoting a significant clinical improvement in terms of UPDRS in all three subjects [77].

The summary of clinical trials of MSC therapy for PD is presented in Table 2.

**Table 2:** The summary of clinical trials MSC application in the treatment of PD

Action and target cell	Investigators	Year and location	Estimated enrollment	Purpose	Status	Trial phase	Clinical Trials ID
Intravenous administration of autologous bone marrow derived MSCs	Yang Xiao Li Li	2011 Guangzhou General Hospital of Guangzhou Military Command	20	Safety and efficacy	Unknown	Phase 1/2	NCT01446614
Intra-arterial administration of autologous bone marrow derived MSCs in progressive supranuclear palsy	Rosaria Giordano	2012 Fondazione IRCCS Ca' Granda, Ospedale Maggiore Policlinico	25	Safety and efficacy	Unknown	Phase 1/2	NCT01824121
Intra-arterial and intravenous administration of autologous adipose-derived SVF cells	Sharon McQuillan	2014 Ageless Regenerative Institute	0	Safety and efficacy	Recruiting participants	Not Applicable	NCT01453803
Autologous adipose-derived SVF cells. Route of administration not defined	StemGenex	2014 StemGenex San Diego	75	Safety and efficacy	Unknown		NCT02184546
Intravenous administration of allogeneic bone marrow-derived MSCs	Mya Schiess	2017 The University of Texas Health Science Center, Houston	20	Safety, feasibility, and efficacy	Recruitment completed	Phase 1	NCT02611167
Intravenous administration of Umbilical Cord Derived MSCs	Xiqing Chai	2018 Hebei Newtherapy BIo-Pharma Technology Co., Ltd	20	Safety and efficacy	Enrolling by invitation	Phase 1	NCT03550183
Intrathecal and Intravenous administration of umbilical cord derived MSCs	Abdallah Awidi	2018 University of Jordan	10	Short term and long term safety	Recruiting	Phase 1/2	NCT03684122
Infusion of allogeneic bone marrow-derived MSCs	Mya C Schiess	2020 The University of Texas Health Science Center, Houston	45	Safety and efficacy	Not yet recruiting	Phase 2	NCT04506073

## Exosomes; Act as MSC paracrine mediators

### Definition of exosome

Exosomes are EVs derived from the endosomal complex [78]. EVs are divided into three categories based on their size, cargo, and origin: exosomes, microvesicles, and apoptotic bodies [79, 80]. Among EVs, exosomes are the smallest group; they have a size of 30 to 150 nm and can be extracted by centrifugation from all body fluids such as breast milk, blood, urine, amniotic and synovial fluid, ascites, and pleural effusions [81]. Exosomes are secreted by most cell lines, including neurons, immune cells,  epithelial and endothelial cells [82], and have cytosolic substances and phospholipid bilayer that are similar to those of the donor cells [83]. In addition to creating stability and durability of the membrane, the exosome lipid bilayer membrane has a role in other activities such as absorption and fusion by the target cells. Phosphatidylserine (PS), for example, is involved in exosome sprouting and merging due to its enhanced flexibility [84]. Lysosomal-associated membrane protein (LAMP), tetraspanins (CD81, CD82, CD9, CD63), GTPases, major histocompatibility complex-I and II (MHC I-II), CD13, intercellular adhesion molecule-1 (ICAM-1), fusion proteins such as tumor susceptibility gene 101 protein (TSG101), annexin, integrin, heat shock protein 90 (HSC90) and HSC70 are other molecules found abundantly in exosomal membranes [22, 85–87]. These cytosolic-loaded membrane proteins function as biomarkers for a variety of clinical diseases. Exosomes include a specific collection of protein groups from intracellular domains such as the plasma membrane, endosomal pathway, and cytoplasm [78]. Messenger RNAs (mRNA) and microRNAs (miRNA) are also found in exosomes and can carry genetic information to target cells [88].

### Exosomal nucleic acids

**Fig. 1:**
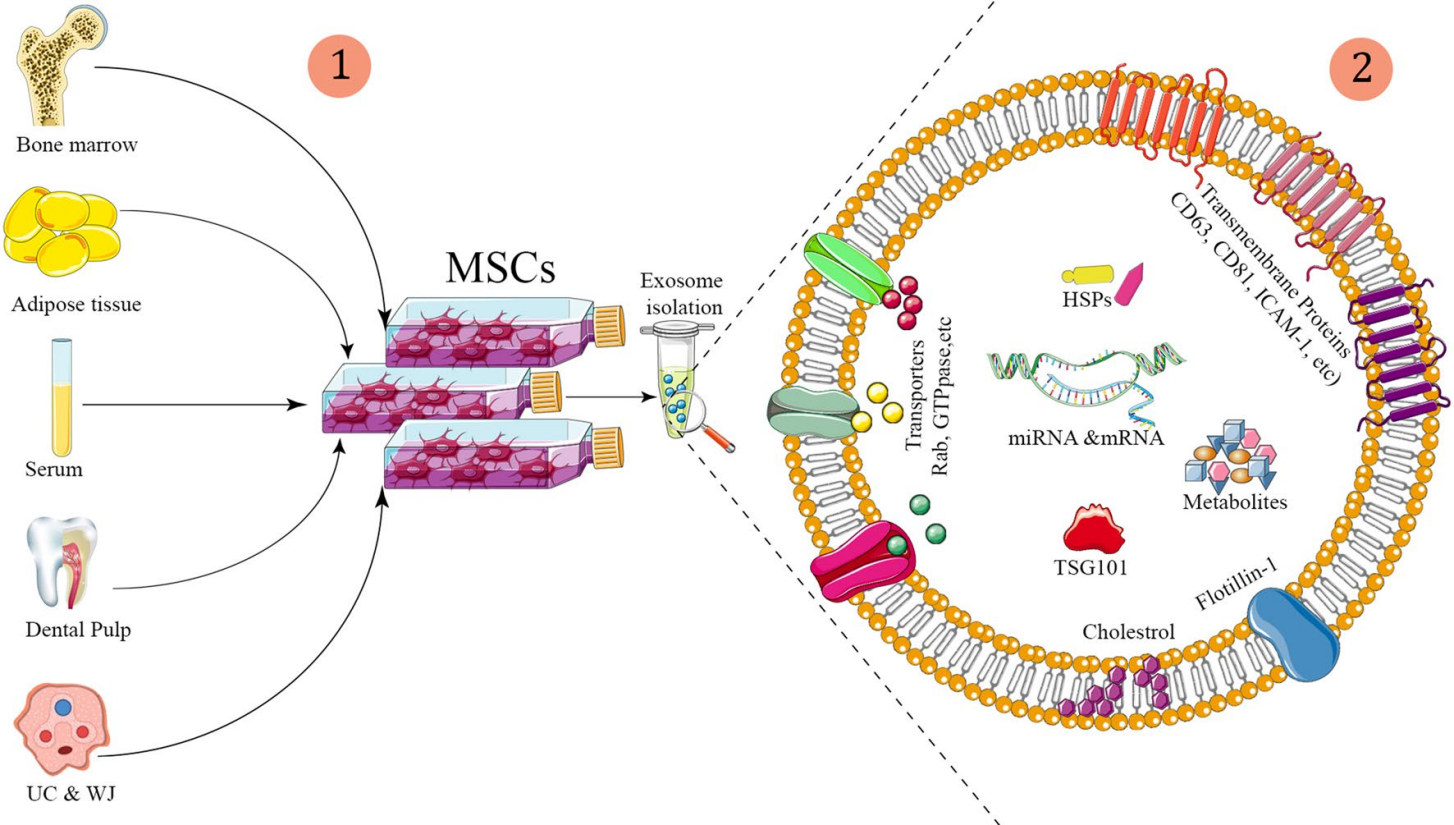
(1) Exosomes are separated from cell culture and bodily fluids using a variety of techniques. (2) They contain a series of Rab proteins, tetraspanins, heat shock proteins, intercellular adhesion molecule (ICAM-1), endosome-associated proteins (TSG101), and nucleic acids such as mRNAs and mRNAs, according to fluorescence-activated cell sorting (FACS), mass spectrometry analyses, and Western blot

As explained previously, nucleic acids like miRNAs constitute a significant component of exosomes [89]. Several disorders, including PD, have been shown to involve alterations in gene expression, particularly at the miRNA stage [90]. MiRNAs generated from exosomes have also been identified as possible diagnostic markers and targeted therapeutics (Fig. 1).

MiRNAs are a type of non-coding RNA that plays a crucial role in gene expression regulation. MiRNAs often interact with the 3′ untranslated region (3′-UTR) of target mRNAs to cause mRNA degradation and translational inhibition. However, miRNAs have been shown to interact with other areas such as the 5′-UTR, coding sequences, and gene promoters. MiRNAs can also initiate translation or regulate transcription under certain situations [91].

In animal cells, miRNAs are generated in two phases, beginning with primary miRNAs and continuing with Drosha/DGCR8 RNase in the nuclei and Dicer RNase in the cytoplasm [92]. It was discovered that Dicer deficiencies in the midbrain rats might result in progressive destruction of DAn. Also, post-mortem cerebral examination revealed DAn loss and Lewy body formation when the DiGeorge syndrome critical region 8 (DGCR8) gene was eliminated [93, 94]. Exosomes seem to assist exogenous miRNA distribution to target cells. Regardless, the processes of target absorption should be thoroughly investigated [95].

## MSC-derived exosome characteristics

Having established the definition of exosomes as nanoscopic particles derived from MSCs, one might conclude that they are, in effect, the mediators of MSC functions in a paracrine fashion [96]. Similar to other exosomes, MSCs-derived exosomes transport a complex cargo of lipids, proteins, and nucleic acids. MSC-derived exosomes are particularly suitable for this role due to their high enzyme content [97]. This is because many enzymes' catalytic activity is controlled by feedback mechanisms influenced by the tissue microenvironment. By supplying catalytically active enzymes to support tissue homeostasis, MSC-derived exosomes might be able to restore normal tissue function to some extent, though, such speculations should be validated by prospective investigations.

The activity of an enzyme is proportional to the relative concentrations of its substrate and product, and it stops when the concentrations of its substrate and product are in equilibrium. Because an injury-induced change in the relative concentrations of substrate and product is expected to be proportional to the degree of the injury, exosomes might construct a measurable biological response proportionate to the severity of the injury. In contrast, improving the damage restores substrate and product balance, resulting in the termination of enzyme activity. As a result, the enzyme-centric nature of exosomes reduces the possibility of over-or under-dosing [98].

Exosomes produced from MSCs have also been found to contain cytokines and growth factors such as IL-6, IL-10, HGF and TGFβ-1; all of them have a role in regulating the immune system [99]. Comparable quantities of extracellular matrix metalloproteinase inducer (EMMPRIN), Matrix Metalloproteinase-9 (MMP-9) and VEGF have been found in MSC-derived exosomes; all of which are important in inducing angiogenesis, which may be essential for tissue repair [100]. Many miRNAs have been discovered in MSC-derived exosomes, and they are thought to be involved in physiological and pathological pathways like organism development, epigenetic regulation, immunoregulation (miR-155 and miR-146) [101], carcinogenesis, and tumor growth [102].

MSC-exosomes have been studied for their immunomodulatory characteristics [103]. Exosomes produced by BM-derived MSCs can effectively treat chronic graft-versus-host disease (cGVHD) in mice by suppressing CD4^+^ T cell activation and infiltration, lowering pro-inflammatory cytokine production, enhancing the formation of IL-10-expressing Treg cells, and suppressing Th17 cells [104]. In animal models of type 1 diabetes (T1D) and experimental autoimmune uveoretinitis, EVs derived from human multipotent stromal cells decrease autoimmunity. EVs inhibited antigen-presenting cell (APCs) activation and suppressed the growth of T helper 1 and T helper 17 cells, as well as increasing the immunosuppressive cytokine IL-10 [105].

**Fig. 2:**
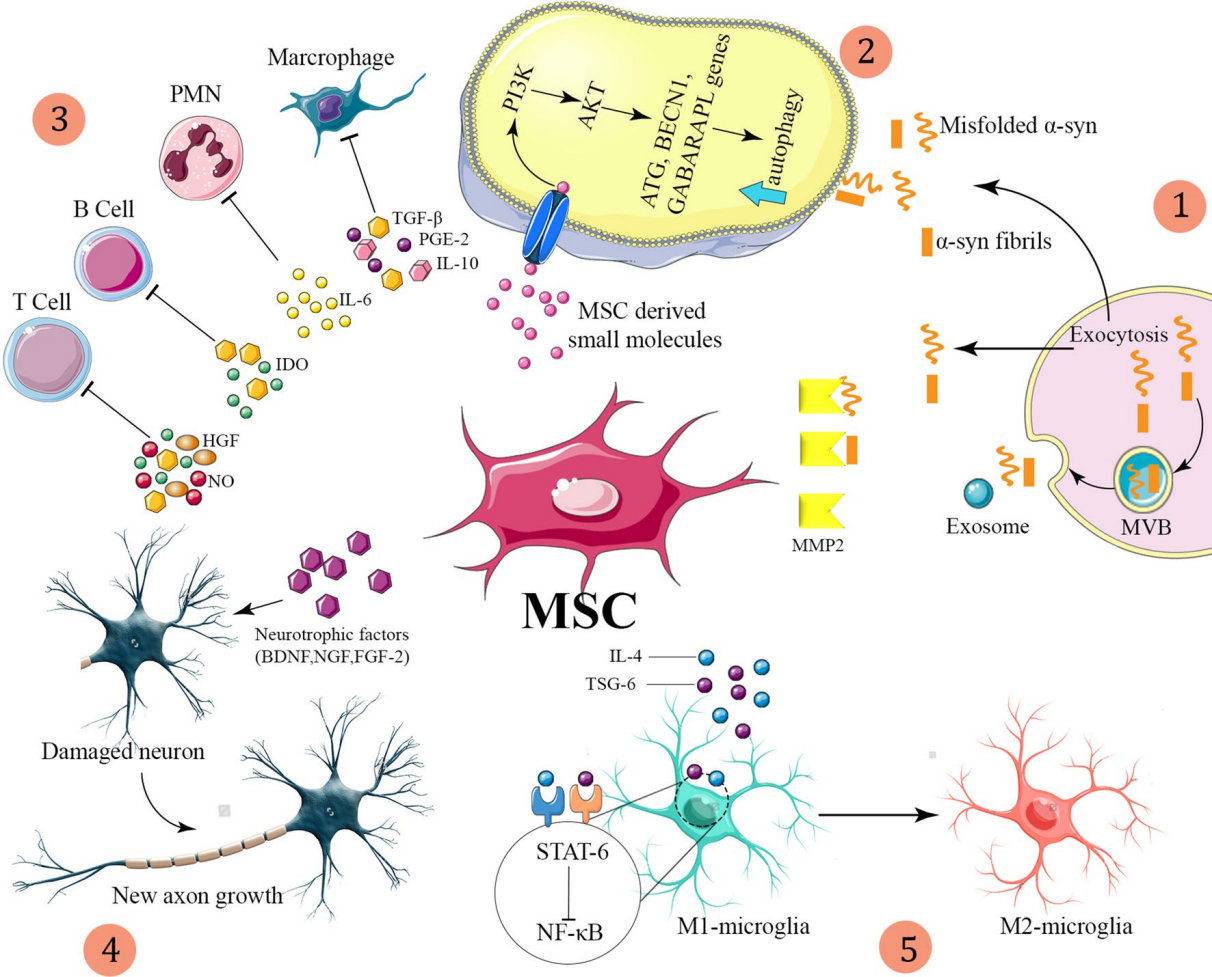
(1) Secretion of α-syn can occur via secretory lysosomes (exocytosis), microvesicle shedding, or multivesicular bodies, with the second and third methods including the release of α-syn into exosomes. α-Syn can be eliminated from the extracellular space by proteolysis. One of the tiny molecules implicated in the proteolytic degradation of aggregated α-syn would be MMP2, a factor generated from MSCs. MMP-2 produced from MSCs breaks freshly formed amyloid fibrils, resulting in a considerable decrease in the quantities of insoluble and oligomeric α-syn. (2) Furthermore, tiny compounds generated from MSCs alter PI3K/Akt signaling, which ultimately regulates multiple downstream targets to increase autophagy. Upregulation of PI3K/AKT promotes autophagy via regulating the expression of autophagy-related genes such as BECN1, ATG, and GABARAPL1. As a result, autophagic flux upregulation by MSC derived small molecules increases the clearance of harmful α-syn aggregates and so plays a vital role in maintaining α-syn homeostasis in the PD-related milieu. (3) MSC interactions with immune system cells, with primary signaling pathways revealed. (4) MSCs secrete neurotrophic factors like as BDNF, NGF, and FGF-2, which interact with injured axons and cause axonal regrowth. 5) When activated by pro-inflammatory mediators, MSCs release paracrine factors such as TSG-6 and IL-4. Paracrine factors stimulate M2 macrophage polarization, resulting in an elevated Th2 response

BMSCs-derived exosomes increases Treg proliferation and immunosuppressive capacity in asthmatic patients' peripheral blood mononuclear cells (PBMCs) via up-regulation suppressive cytokines TGF-1 and IL-10 [106]. hUC-MSC-derived exosomal MiR-181c have an important role in anti-inflammatory activities in a burnt rat model via inhibiting the TLR-4 signaling cascade [107]. Furthermore, MSC-derived exosomes suppressed complement-mediated destruction of sheep red blood cells in a CD59-dependent way, implying that CD59 on the exosome membrane might reduce complement activation and hinder the generation of the membrane attack complex [97]. These findings demonstrate MSC-derived exosomes' immunomodulatory activity and support their potential to restore immunological homeostasis in a tissue (Fig. 2).

### Exosomes’ advantages in PD

There is currently no accurate diagnostic method for PD. Exosomes play two role in PD: diagnostic markers and therapeutic agents. Elevated leucine-rich repeat kinase 2 (LRRK2) mutations in urine have recently been linked to idiopathic PD and the intensity of cognitive impairment [108–110]. Another research revealed that the concentration of the L1 cell adhesion molecule (L1-CAM) was much more in PD patients and that it was linked with a-synuclein level and tau concentrations in cerebrospinal fluid (CSF) [111–114]. The expression patterns of mRNA and miRNA in PD exosomes act as a diagnostic method for the disease. For treatment, exosomes generated from DPSC have been discovered to prevent 80% of dopamine neuron death in PD models [115]. Also, a recent study on a PD model demonstrated that exosomes produced by MSCs could retain human brain microvascular endothelial cells (HBMECs) in a transcriptionally active mode, which may be favorable for angiogenesis [116]. In conclusion, the use of exosomes in the treatment of PD is still in its initial phases, but they are largely applied in diagnosis. Although no reliable exosome-based diagnostic tool is currently available for PD.

## Challenges of stem cell therapy

### MSCs source

The primary source of stem cells is a critical subject to address in order to minimize an immunogenic reaction, especially if persistent immunosuppressive therapy is to be prevented. Because of the minimal risk of rejection, autologous stem cells would be ideal for grafting. However, one negative aspect of autologous transplants is that grafted cells may keep the genetic abnormalities or risk factors resulting from the onset of PD [117]. Though, regardless of the potential immune reactions that might ensue transplantation, isolation of MSCs from candidate donors, based on the source tissue, can be a laborious task. For instance, less than 0.01% of the cells residing in bone marrow are MSCs, and this astonishingly low prevalence makes their isolation considerably difficult [118], particularly when one considers the fact that bone marrow aspiration is an invasive and painful procedure, and not all donors would opt to participate in such procedure. In this sense, adipose tissue might provide a better source for extraction of MSCs, as it is not as deeply located as the bone marrow, is more abundantly distributed throughout the body and can be accessed through less invasive methods [119].

### Graft location

Several methods are currently available, either experimentally or practically, for transplantation of MSCs. In a more general sense, MSCs can be administrated either systemically or locally. Systemic transplantation is achieved via intravenous/intra-arterial infusion or inhalation of MSCs. While systemic transplantation allows MSC-based treatment of pathologies affecting the entire body, local transplantation aims to alleviate symptoms associated with illnesses that originate from certain organs, and is mostly performed through intramuscular or direct tissue injection. Each of these are limited in their own field [120].

The dopaminergic neurons are predominantly depleted in PD in the substantia nigra pars compacta (SNpc). These neurons send dopamine to the receiving cells via the putamen and caudate nuclei. As a result, several SNpc cell therapy trials in animals transplant dopaminergic neurons ectopically into striatums [121, 122]. However, since the human neural network is significantly larger than rat brains, the size of neuronal extensions may still be insufficient to stimulate the striatum if the cells are transplanted in the SNpc. As a result, transplanting into the putamen, where dopaminergic neurons finally innervate, is still the best option.

### Graft cell count

Several studies have demonstrated that stem cell-derived dopaminergic transplants persist in animals after graft for at least 2 years. Still, fetal-derived dopaminergic transplants have persisted in human hosts for more than a decade. Several investigations have found that a grafting could be a suitable method for the long-term survival and functioning of transplants [122–124]. In the case of dosing, the average dose of each MSC therapy session in experimental animal models is usually 50 million cells per every kg of weight. Though, the baseline dose in human studies is 1–2 million cells/kg and rarely exceeds 12 million MSCs/kg [125]. According to a 2020 systematic review by Katab et al., intravenous infusion of MSCs is the most commonly preferred route of transplantation practiced in 43% of all clinical trials on MSC therapy, with every single dose per patient containing a median count of 100 million MSCs. Intravenous infusion of as many MSCs has often been deemed as safe and effective, rarely resulting in adverse effects beyond transient fever [126].

### Removing the risk of neoplasia

When implanted into patients, PSCs resistant to differentiation stimuli poses an increased neoplasia risk [127]. Multiple techniques for mitigating this danger have been developed, broadly classified as removing stem cells from ultimate cultures and purifying the target cell types. For example, undifferentiated PSCs exhibit distinct cell surface biomarkers [128]. Although this "negative-sorting" technique has effectively removed undifferentiated PSCs in a mixed culture [129].

Moreover, in order to make stem cells more clinically reliable, the process by which stem cells function in experimental animals should be properly known, and patients would be able to use stem cell therapy instead of expensive drug treatment when faced with organ failure [130]. Also, bioengineering methods, such as loading MSCs with oncolytic viruses and recombinant genes, can be employed to address the challenges mentioned earlier and provide more effective and predictable MSC therapies [131–133].

## Advantages of MSCs over other stem cell lines

PD is a devastating neurological condition that affects millions of individuals worldwide; yet, the molecular and cellular pathways that cause it are still unknown. Despite improvements in Parkinson's disease research, current pharmaceutical options improve PD patients' quality of life but do not prevent PD progression or boost dopaminergic neuron viability. Because of their capacity to promote dopaminergic neurons viability, stimulate neurogenesis, reduce neuroinflammation, and enhance functional recovery in the in vivo models, cell therapy with MSCs and their derived exosomes has recently been proposed as an efficient treatment method for a variety of neurodegenerative disorders, including PD.

In any case, investigations involving MSC and PD point to the possibility that these cells could be used as a standard treatment in the coming years. The ability of MSC to differentiate into diverse types of neurons shows that MSC can replace damaged cells not just in the basal nuclei and nigrostriatal pathway but also in other parts of the parenchyma whose degradation leads to the cognitive and behavioural symptoms of PD [134]. However, this is not the only advantage of MSCs. In fact, these cells are inert in terms of immunogenicity, as their progeny do not include immune cells, in contrast to hematopoietic stem cells (HSCs), for example. In addition to this, MSCs can be safely suppressed when required by immune cells, a capability that minimizes their potential hazards to considerable extents. Owing to their lineage, MSCs have also been shown to facilitate engraftment of other stem cells, indicating that they are quite good housekeepers. This is further bolstered by the fact that these cells can be maintained for prolonged periods of time in their initial state without transformation [135]. Else, MSCs bring about salutary alterations in their surrounding environment by releasing an extensive secretome including extracellular vesicles, especially miRNA-containing exosomes that participate in intercellular signal transduction, which has been found to confer neurorestorative effects in the case of ischemic stroke [136]. By secreting these exosomes, MSCs facilitate cell proliferation and angiogenesis, while repressing apoptosis, all of which are of high therapeutic value in degenerative diseases, such as PD [137].

## Conclusions

Although stem cells continue to be an invaluable treatment modality for neural regenerative medicine, their therapeutic use is limited by the possibility of immune reactivity, tumorigenesis, and insufficient differentiation, as well as non-specific targeting and the inability to cross physiological and biological barriers. MSC-derived exosomes not only have therapeutic characteristics similar to their parent cells, but they also have the ability to avoid whole-cell post-transplant adverse events due to their potential to pass physiological barriers, migrate and reside in brain lesion sites, have a high safety profile, and no cases of immune response and rejection have been reported. Furthermore, exosomes cannot turn into pre-malignant cells. In animal models, the exchange of genetic material, such as miRNA, via exosomes can enhance neurogenesis, reduce neuroinflammation, and promote functional recovery. Indeed, miRNAs have acquired prominence in the PD research area, not only for their role in PD pathogenesis but also as a promising window for usage as biomarkers or potential therapeutic agents for PD treatment.

Nonetheless, one should not overlook the experimental nature of such therapeutic interventions, as the majority of clinical trials concerned with MSC therapy, particularly in the case of PD, have had few participants, and the results obtained from these initiatives cannot necessarily be generalized to everyday medical care. In addition to this, isolation and transplantation of MSCs require resource-demanding methods that are usually costly, and there is always a risk of failure, even if low, that need to be considered.

## Data Availability

The data supporting the conclusions of this article are all online.

## Abbreviations

PD: Parkinson's disease
DAn: Dopaminergic neurons
SN: Substantia nigra
MSCs: Mesenchymal stem cells
ESCs: Embryonic stem cells
iPSCs: Induced pluripotent stem cells
NSCs: Neural stem cells
EVs: Extracellular vesicles
UCHL1: Ubiquitin carboxy-terminal hydrolase L1
PINK1: Putative serine-threonine kinase
BCEN1: Beclin 1 gene
GABARAPL1: Gamma-aminobutyric acid receptor-associated protein-like 1
ATG12: Autophagy-related gene 12
TGF-β1: Transforming growth factor-beta 1
PGE2: Prostaglandin E2
HGF: Hepatocyte growth factor
IDO: Indoleamine 2,3 dioxygenase
NO: Nitric oxide
IL-4: Interleukin 4
IL-10: Interleukin 10
IL-6: Interleukin 6
IL-1β: Interleukin-1β
TNF-α: Tumor necrosis factor-alpha
GDNF: Glial cell-derived neurotrophic factor
NGF: Nerve growth factor
BDNF: Brain-derived neurotrophic factor
VEGF: Vascular endothelial growth factor
FGF2: Fibroblast growth factor 2
BMSCs: Bone marrow-derived mesenchymal stem cells
6-OHDA: 6-Hydroxydopamine
ASCs: Adipose stem cells
SVZ: Subventricular zone
TH: Tyrosine hydroxylase
DPSC: Human dental pulp stem cells
CJ-MSCs: Human conjunctiva mesenchymal stem cells
UPDRS: Unified PD Rating Scale
S&E: Schwab and England
H&Y: Hoehn and Yahr
LAMP: Lysosomal-associated membrane protein
MHC I,II: Major histocompatibility complex I, II
ICAM-1: Intercellular adhesion molecule-1
TSG101: Tumor susceptibility gene 101 protein
HSC90: Heat shock protein 90
HSC70: Heat shock protein 70
mRNA: Messenger RNA
miRNA: MicroRNA
3′ UTR: 3′ untranslated region
DGCR8: DiGeorge syndrome critical region 8
APCs: Antigen-presenting cells
DCs: Dendritic cells
LRRK2: Leucine-rich repeat kinase 2
L1-CAM: L1 cell adhesion molecule
CSF: Cerebrospinal fluid
HBMECs: Human brain microvascular endothelial cells
SNpc: Substantia nigra pars compacta
EMMPRIN: Extracellular matrix metalloproteinase inducer
MMP-9: Matrix metalloproteinase-9
PBMCs: Peripheral blood mononuclear cells
